# Genome-wide association studies reconstructing chronic kidney disease

**DOI:** 10.1093/ndt/gfad209

**Published:** 2023-12-20

**Authors:** Anastasios Fountoglou, Constantinos Deltas, Ekaterini Siomou, Evangelia Dounousi

**Affiliations:** Department of Nephrology, Faculty of Medicine, School of Health Sciences, University of Ioannina, Ioannina, Greece; School of Medicine and biobank.cy Center of Excellence in Biobanking and Biomedical Research, University of Cyprus, Nicosia 2109, Cyprus; Department of Pediatrics, Faculty of Medicine, School of Health Sciences, University of Ioannina, Ioannina, Greece; Department of Nephrology, Faculty of Medicine, School of Health Sciences, University of Ioannina, Ioannina, Greece

**Keywords:** chronic kidney disease, genetic kidney diseases, genetic variants, genome-wide association studies (GWAS)

## Abstract

Chronic kidney disease (CKD) is a major health problem with an increasing epidemiological burden, and is the 16th leading cause of years of life lost worldwide. It is estimated that more than 10% of the population have a variable stage of CKD, while about 850 million people worldwide are affected. Nevertheless, public awareness remains low, clinical access is inappropriate in many circumstances and medication is still ineffective due to the lack of clear therapeutic targets. One of the main issues that drives these problems is the fact that CKD remains a clinical entity with significant causal ambiguity. Beyond diabetes mellitus and hypertension, which are the two major causes of kidney disease, there are still many gray areas in the diagnostic context of CKD. Genetics nowadays emerges as a promising field in nephrology. The role of genetic factors in CKD’s causes and predisposition is well documented and thousands of genetic variants are well established to contribute to the high burden of disease. Next-generation sequencing is increasingly revealing old and new rare variants that cause Mendelian forms of chronic nephropathy while genome-wide association studies (GWAS) uncover common variants associated with CKD-defining traits in the general population. In this article we review how GWAS has revolutionized—and continues to revolutionize—the old concept of CKD. Furthermore, we present how the investigation of common genetic variants with previously unknown kidney significance has begun to expand our knowledge on disease understanding, providing valuable insights into disease mechanisms and perhaps paving the way for novel therapeutic targets.

## INTRODUCTION

Chronic kidney disease (CKD) affects more than 850 million people [1] and is a global public health problem with significant impact on morbidity and mortality rates [2], and increasing economic and social burden mainly attributable to the care of end-stage renal disease (ESRD) patients [3]. Unfortunately, care of CKD patients has many problems in the fields of diagnostics and therapeutics, the most important of which can be attributed to the lack of a precise diagnosis in many circumstances, suboptimal diagnostic workup and the ignorance of the main biologic mechanisms that drive kidney injury. Diagnosis such as CKD of unknown cause, focal segmental glomerulosclerosis, hypertensive nephropathy and diabetic kidney disease constitute inaccurate and confusing diagnostic labels in many cases [4], giving rise to many gray area in the classification system of chronic nephropathy.

In recent years, new methods of genetic analysis have allowed clarification of some of these gray areas, highlighting genetic forms as an important, although underestimated, cause of CKD. In this review we aim to address the two major categories of genetic kidney disease, namely monogenic/Mendelian and polygenic, and focus on the genetic predisposition of CKD and how this is beginning to be delineated through genome-wide association studies (GWAS).

## GENETIC ARCHITECTURE OF CKD

Genetic investigation of patients with CKD and detection of many rare Mendelian kidney diseases has revolutionized the concept of chronic nephropathy, giving rise to systematic and thorough research in the field of kidney genetics (nephrogenetics). Nevertheless, the impact of such monogenic diseases on the high epidemiological burden of CKD remains low, providing evidence that other genetic factors may contribute to the increasing prevalence of CKD in the general population incorporated in a more complex genetic context.

In the case of CKD, genetically influenced kidney diseases consist of a wide spectrum that extends from monogenic kidney diseases, in which a pathogenic variant in a single gene is necessary and sufficient to produce a pathological kidney phenotype, to a polygenic model of kidney disease where the co-inheritance of multiple different gene allelomorphs are necessary to cause a disease. In many cases of multifactorial conditions, multiple genetic variants may be necessary but not sufficient to manifest a phenotype, as the environment also has a variable contribution.

Monogenic nephropathies, caused by pathogenic variants in single genes, are now known to be an important, albeit underappreciated, cause of CKD, with research data so far from various studies estimating that they are responsible for 70% and 10%–15% of ESRD cases in children and adults, respectively [5–7]. It is also characteristic that these monogenic causes explain approximately half of the cases of familial steroid-resistant nephrotic syndrome, congenital tubulopathy and atypical hemolytic uremic syndrome, lengthening the distance to the complete clarification of the genetic basis of the kidney disease [8]. To date, more than 600 single-gene causes of CKD have been identified [9], which involve all structures of renal parenchyma (glomeruli, renal tubules, interstitial space) including both independent non-syndromic and syndromic forms of kidney disease.

Polygenic nephropathy, on the other hand, refers to the genetic predisposition of kidney disease, either in the context of a known disease entity with a high nephrotoxic potential [e.g. diabetes mellitus (DM)] or in other disease contexts with varying degrees of clinical presentation. This predisposition is multifactorial in nature, with an important genetic component. To date, hundreds of genetic loci and single nucleotide polymorphisms (SNPs) have been identified, associating with traits of renal function such as glomerular filtration rate (GFR) and albuminuria as well as with specific entities of CKD [10–16].

Nevertheless, the distinction between monogenic diseases with clear causal relationship between genotype and phenotype, and an oligo/polygenic pattern of kidney damage in which co-existence of many common pathogenic alleles is required, possibly in interaction with other factors, constitutes a rather simplified approach to a more complex reality that simply satisfies the needs of a theoretical typology. In fact, genetic variants involved in kidney damage constitute a wide genetic spectrum, the extremes of which extend from rare variants [minor allele frequency (MAF) <1%] with high penetrance (100%) causing distinct disease entities to more frequent variants (MAF >5%) with low penetrance that participate in polygenic patterns of renal damage, which have emerged recently with GWAS (see below).

**Figure 1:**
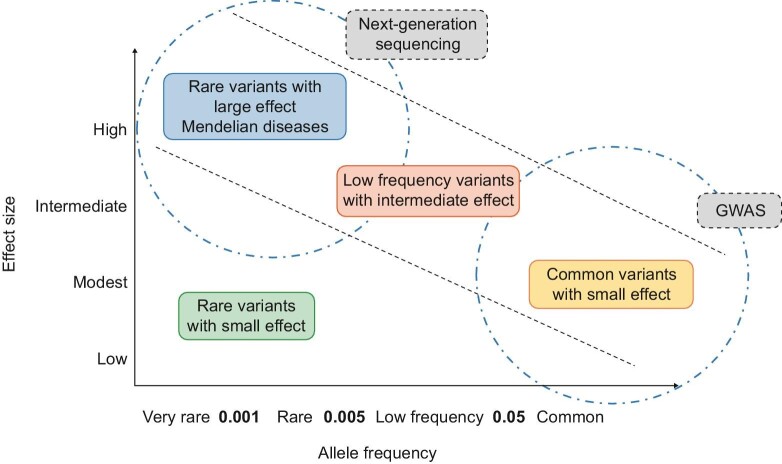
Genetic architecture of chronic kidney disease.

The genetic architecture of CKD (Fig. 1) is completed by variants with MAF between 0.5% and 5% and intermediate penetrance or rare genetic variants with MAF <0.5% and low penetrance [17]. These variants cannot be detected through GWAS studies, while their low impact on phenotype make them “invisible” to genetic DNA linkage analysis in families.

However, genetic variants that have been identified to date fail to explain the entire genetic predisposition to kidney disease, leading researchers to formulate the theory of lost heritability. In fact, monogenic causes of CKD explain only 10% of total CKD cases [4] while common alleles that emerge with GWAS studies explain only 20% of impaired GFR heritability [18].

## GWAS IN CKD

Implementation of new methods of genetic analysis such as GWAS has given a great boost to the field of nephrogenetics, revealing new genetic loci and implicating previously unknown disease mechanisms as causative of kidney damage. These studies constitute mapping methods that identify genetic variants associated with an outcome across the genome in an unbiased manner. With GWAS, researchers do not focus on specific genes but screen the entire genome in search of a statistical association between a genotype, typically an SNP, and the outcome, typically a kidney marker or disease.

Finding an SNP in a patient group that shows a significantly different frequency from that in the control group identifies a candidate genetic locus that is likely associated with the phenotype under consideration. The reverse finding signifies a SNP with a potential protective effect. This finding does not automatically translate into establishing a causal relationship between the particular variant and the phenotype. On the contrary, in most cases this association is observed due to linkage disequilibrium between the genetic variant in question and the gene alleles that truly contribute to the pathogenesis of a phenotype.

GWAS have allowed the discovery of dozens of significantly associated and highly reproducible genetic loci [10–16], leading to functional studies in model organisms or cell lines in order to validate causal genes or variants [15]. The two largest GWAS, which were performed recently by Wuttke *et al*. [18] and Stanzick *et al*. [19] in more than 1 000 000 individuals, revealed the association of 264 and 424 genetic loci, respectively, with eGFRcreatinine (eGFRcre). With the incorporation of other biomarkers, such as cystatin C and urea, the number of aforementioned genetic loci was limited to 147 and 348, respectively. This methodological approach excludes genes involved in creatinine metabolism, whereas it allows the identification of genes that are truly associated with CKD and markers of kidney damage.

## FROM GWAS TO ETIOPATHOGENESIS OF CKD

GWAS attempt to resolve a major existential problem of nephrologists worldwide: the largely ignored biological mechanisms behind CKD which in practice translates into a lack of therapeutic targets and effective treatments. With GWAS, knowledge about the pathogenesis of CKD gradually began to enrich, allowing the identification of genetic loci with possible implication in kidney damage mechanisms.

An important challenge of GWAS is the translation of the above genetic associations into specific causal genes and understanding how these affect critical biological processes in the kidneys. The vast majority of these genetic variants are located in non-coding regions of the genome, which underscores the complexity of gene expression. The classic Mendelian view of genetic diseases, according to which a gene variant causes disease through its direct effect on the coding of the corresponding protein, is far from the multi-gene model of kidney disease, where the interplay of many DNA variants with minor effects is necessary for the expression of a given phenotype. Additionally, it seems that many of these variants are located in regulatory gene regions, where they change the binding affinity of transcription factors and therefore the degree of expression of contiguous or distant genes [20]. In addition, other variants exhibit significant cell-specificity in their expression, making the whole process of identification of the responsible genes particularly difficult [20].

Based on these limitations, contributory variant and cell-type prioritization has become a crucial post-GWAS field of interest. Using advanced statistical and computational methods scientists try to interpret GWAS results in order to identify the exact disease-causal variants, the genes they regulate and the cell types in which these variants are active. A gold standard practice for the prioritization of target genes is the combination of data from GWAS, expression quantitative trait loci (eQTL) datasets and single-cell gene expression analyses, providing the opportunity for a thorough experimental validation and follow-up [21]. These methods might allow scientists to move from genetics to gene functions and disease biology, in order to identify the exact drivers of kidney disease biology and to establish future therapeutic targets.

Although our current knowledge about these genes is quite limited, we nevertheless know with relative certainty that these variants are not related to major kidney disease risk factors, such as DM and arterial hypertension (AH), but with kidney function *per se*. Therefore, the induction of damage seems to be mediated through a possible gradually increasing vulnerability of the renal parenchyma to these nephrotoxic risk factors [16].

Additionally, in contrast to the hundreds of genetic loci associated with GFR, there are few that differ between diabetic and non-diabetic CKD patients, suggesting a common genetic predisposition to renal dysfunction among those patients that operates independently of DM [22].

The majority of SNPs identified by GWAS pertain genes involved in nephrogenesis, in structural and functional integrity of the glomerular filtration barrier and podocyte function, in angiogenesis, in tubular transport, in kidney metabolism and in the function of tubular primary cilia [12]. At the cellular level many of them participate in gene transcription and cell signaling and differentiation [23], revealing a complex framework of genetic predisposition. Due to this predisposition, multiple “small” deficits in the structural and functional integrity of kidney make the latter particularly vulnerable to the major “stress” factors, such as DM, AH, drugs and immune-induced damage.

Despite the apparent complexity of structural and functional levels of gene expression in the kidney, a significant number of the related genes are located in the tubular compartment, leading to a disease model according to which stress exerted on the renal parenchyma (e.g. by DM, AH and possibly xenobiotics) triggers a common pathogenetic pathway focused on the renal epithelium [24]. It is well established that interstitial damage and development of interstitial fibrosis are common, final patterns of tissue damage in almost all forms of kidney disease, including glomerular diseases, with their extent determining both severity of disease and response to various treatment regimens [25]. It seems that the pathway through which interstitial space responds to an initial stressful stimulus, as well as its capacity to promote and sustain mechanisms of tissue repair, are genetically determined, involving various genes expressed in the individual structures of this space [19, 21].

**Figure 2:**
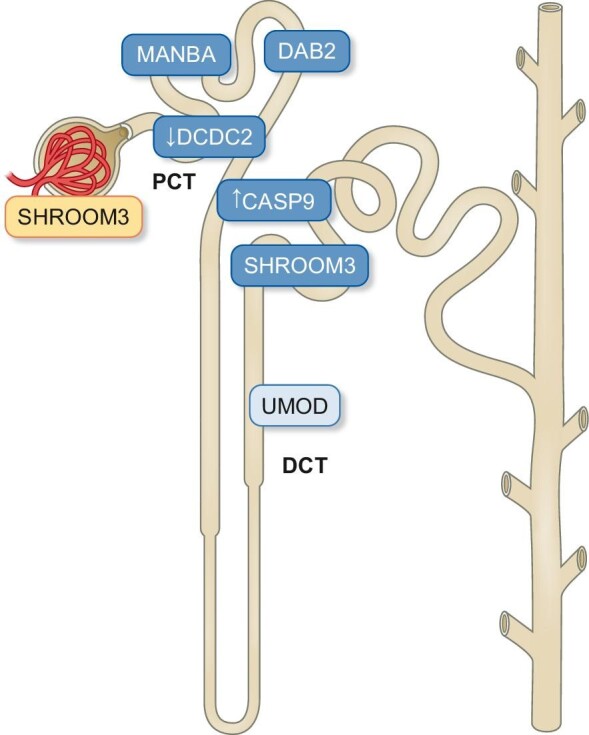
A synopsis of several CKD related gene variants which are expressed mainly at renal tubules level. Among these genes, SHROOM 3 is implicated in both glomerular and tubular renal endophenotypes, giving an excellent example of the cell-specificity of genes expression. SHROOM3, Shroom family member 3.

**Figure 3:**
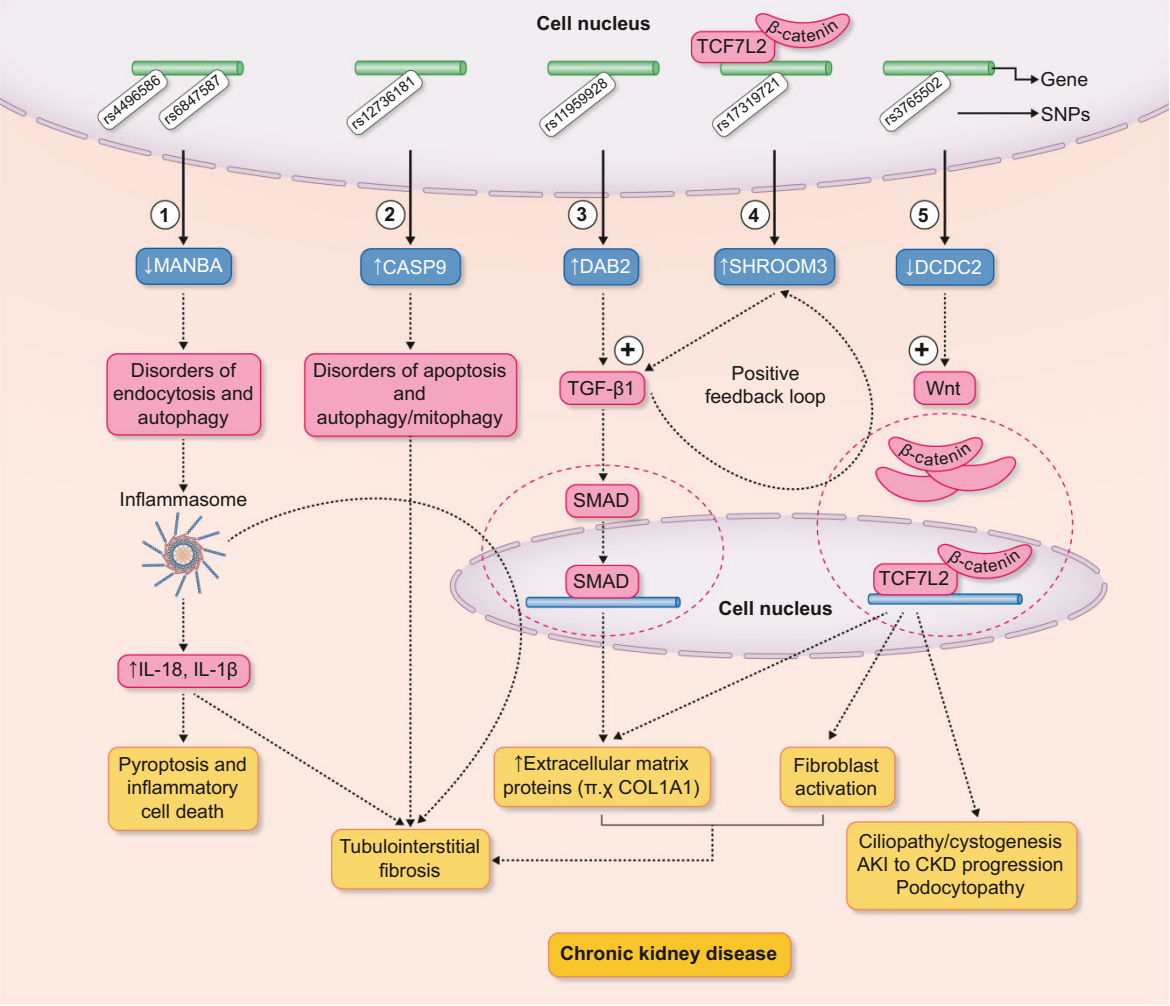
A synopsis of tubular GWAS risk variants and related pathogenic mechanisms: 1. Decreased expression of MANBA is associated with lysosomal dysfunction, disorders of endocytosis and autophagy processes, inflammasome and pyroptosis activation and finally kidney fibrosis; 2. Increased expression of CASP9 is associated with disorders of apoptosis and autophagy/mitophagy processes that are involved in renal fibrogenesis; 3. Increased expression of DAB2 leads to an overactive TGF-β1 signaling pathway through both activation of TGF-β receptors—SMAD proteins and increased clathrin-mediated recycling of TβRII receptors, which protect them from degradation (not seeing in the image); 4. Increased expression of SHROOM3, mediated by a stronger association of TCF7L2/β-catenin transcriptional complex with the corresponding gene, facilitate intracellular TGF-β1 signaling pathway and related fibrotic mechanisms. At the same time TGF-β1 increases SHROOM3 expression through Wnt/β-catenin/TCF7L2 pathway and in this way a positive feedback system is created, promoting renal fibrosis; and 5. Decreased expression of DCDC2 is related with an overactive canonical Wnt signaling pathway which in known to be implicated in many kidney phenotypes: renal fibrosis, progression of AKI to CKD, ciliopathy and cystogenesis, podocytopathy. MANBA, beta mannosidase; CASP9, caspase-9; DAB2, disabled-2; TGF-β1, transforming growth factor beta 1; SHROOM3, shroom family member 3; TCF7L2, transcription factor 7 like 2; DCDC2, doublecortin domain containing 2; AKI, acute kidney injury.

In fact, the majority of GWAS variants associated with kidney function are expressed mainly in the proximal tubular compartment [20, 21], confirming a tubulo-centric view of acute and chronic kidney damage, according to which proximal tubule is the primary target of injury and progression of kidney disease [26] (Fig. 2). Renal proximal tubules showed significant enrichment for GWAS-eQTL target genes and among them, *DAB2, MANBA* and *CASP9* have been prioritized as playing a central role in the fibrotic mechanisms of CKD (Fig. 3). DAB2 is an adaptor protein involved in carcinogenesis as a tumor suppressor, as well as in a plethora of cellular functions such as endocytosis, intracellular signaling, cytoskeleton organization, cell–cell connectivity, etc. [27]. At tubular level DAB2 interacts with transforming growth factor (TGF)-β receptors and SMAD proteins, facilitating their activation, while it also appears to promote the clathrin-mediated recycling of TβRII receptors, protecting them from degradation [28]. Downregulation of *DAB2* gene expression in experimental animals protects them from renal injury and fibrosis possibly through reduced activation of the intracellular TGF-β1/Smad2/3 pathway and reduced expression of pro-fibrotic genes such as *fibronectin* and *collagen 1*. In contrast, the risk allele of *DAB2*, rs11959928, which has been associated with an increased risk of CKD in several GWAS studies [10, 12], leads to a higher expression of DAB2 protein in the proximal tubular compartment and possibly to an increased risk of interstitial fibrosis through the activation of intracellular pathway of TGF-β1 [20].

Likewise, the *MANBA* gene product, a lysosomal β-mannosidase enzyme, is involved in fibrotic mechanisms of tubulointerstitial space and CKD progression through its role in the lysosomal structural and functional integrity. The latter is known to be involved in the pathogenesis of various kidney diseases, with Fabry disease and cystinosis being the prototypes of lysosomal kidney diseases [29]. Low expression of MANBA in experimental models is associated with a greater degree of intertubular damage while frequent non-coding variants on chromosome 4 are related to reduced expression of MANBA in renal tubules and increased risk of CKD [30, 31]. These forms of damage are mediated by lysosomal dysfunction which in turn cause disturbances in endocytosis and autophagy mechanisms, activation of inflammatory processes (inflammasome, pyroptosis), increased production of fibrotic factors and finally establishment of interstitial fibrosis [31], highlighting these damage pathways as potential future targets of kidney therapy.

*CASP9* is another gene that was prioritized as a kidney disease risk gene in proximal renal tubules through its higher tubular expression. The product of *CASP9*, caspase 9, is implicated in apoptosis and autophagy/mitophagy processes and its level of expression is correlated with fibrosis severity [32]. Interestingly pharmacological inhibition of *CASP9* induces a dampened inflammation and lesser fibrosis, opening new horizons for kidney disease therapeutics [32].

**Figure 4:**
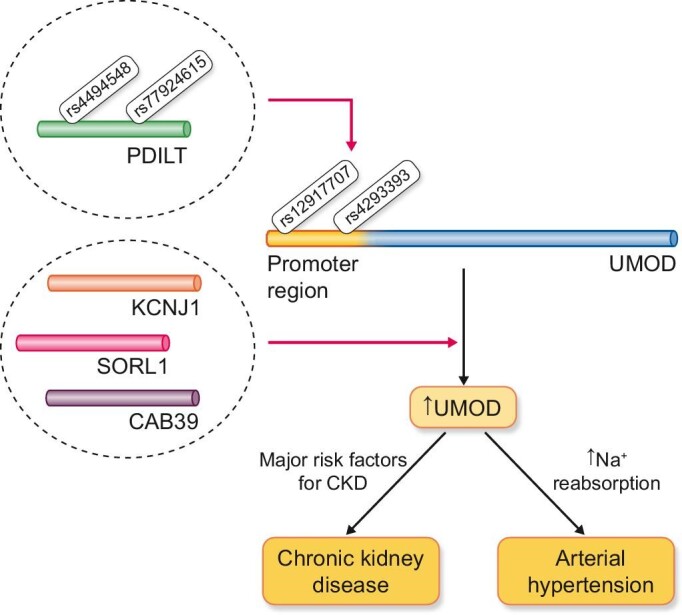
Pathogenic model of UMOD variants related to chronic kidney disease and arterial hypertension: UMOD/PDILT genetic variants increases risk of CKD and arterial hypertension through increased UMOD expression and increased tubular production of UMOD. On the other hand, genes such as KCNJ1/ROMK, SORL1, and CAB39 seems to be correlated to increased UMOD tubular expression through the interactions between transport processes and UMOD secretion in the cells lining the TAL. UMOD, uromodulin; PDILT, protein disulfide isomerase like; KCNJ1/ROMK, potassium inwardly rectifying channel subfamily J member 1; SORL1, sortilin related receptor 1; CAB39, calcium-binding protein 39; TAL, thick ascending limb of the loop of Henle.

Other risk variants found to be strongly associated with CKD in many GWAS are the UMOD risk variants. These variants are mainly expressed in distal convoluted tubules and are associated with higher tubular expression of UMOD. Research interest regarding the UMOD protein has increased since 2009 when Anna Kottgen's group performed one of the first GWAS studies in the field of nephrology demonstrating an association between variants (SNPs) of the *UMOD* gene (rs12917707 and rs4293393) and the risk of developing CKD [10–12]. It is now clear that *UMOD* genetic variants increase the risk of CKD and AH through increased gene expression and increased tubular production of UMOD33. The latter increases activity of luminal cotransporters NKCC2 (Na^+^/K^+^/Cl^–^ cotransporter) as well as potassium channels ROMK (Renal outer medullary potassium channel) in the thick ascending limb of the loop of Henle, explaining the pathogenic basis of AH associated with *UMOD* variants through increased Na^+^ reabsorption [33, 34]. However, the pathway via which increased levels of UMOD causes kidney damage is still questionable. Although hypertension is an attractive mediator of this association, experimental data do not confirm such a correlation. In particular, similar renal lesions are not found in other animal models of hypertension [35], while on the other hand key features of these lesions (e.g. increased levels of Lcn2 and Kim-1) are found in several age-related cases of nephropathy (aging kidney) (Fig. 4). This fact, combined with recent data that association between SNPs of the *UMOD* gene and CKD becomes more pronounced in elderly individuals with other comorbidities [14, 15], allows us to hypothesize a double-hit pathogenic model in which genetic variants of the *UMOD* contributes to the genetic predisposition of renal disease. However, to cause kidney disease, this genetic element requires the co-existence of other major risk factors.

A relative deviation from this tubulo-centric view of CKD is the *SHROOM3* gene, which GWAS variants are related to both glomerular and tubular kidney disease endophenotypes. SHROOM3 is an actin-binding protein that participates in the regulation of epithelial cell architecture through its interactions with actin filaments and microtubules of the cytoskeleton [36]. At kidney level, SHROOM3 is important for the development and maintenance of podocyte integrity through its interactions with actin cytoskeleton both at the apical podocyte surface and at the level of foot processes and slit diaphragms [37]. Specifically, SHROOM3 participates in the activation of ROCK (Rho-associated protein kinase) kinase, which in turn phosphorylates and activates myosin II, leading to the formation of a functional actin–myosin network and the stabilization of the actin cytoskeleton in the apical podocyte region [38]. Its absence causes the disorganization of this network and the disruption of the podocyte morphology and architecture, resulting in the appearance of glomerular damage (sclerosis) and albuminuria. On the contrary, at the level of slit diaphragms, SHROOM3 seems to stabilize the interaction between nephrin and cytoskeleton network through activation of FYN kinase and phosphorylation of the nephrin intracellular domain at the sites of interaction with the NCKap adapter protein [39].

Recent GWAS studies have identified associations between variants of the *SHROOM3* gene and established CKD diagnostic markers [eGFRcre, eGFRcystatin, urine albumin–creatinine ratio (UACR)]. One of these genetic variants is located in the ASD1 domain of SHROOM3, disrupting its interaction with actin and triggering processes of podocyte dysfunction [40]. Moreover, the introduction of a wild-type *Shroom3* allele in fawn-hooded hypertensive (FHH) models has been shown to significantly reduce glomerular damage (podocyte foot process (PFP) fusion, glomerulosclerosis and albuminuria) [40]. These findings support the hypothesis that hypomorphic mutations of the *SHROOM3* gene in humans could lead to kidney damage or increased genetic predisposition to kidney disease in the context of other non-genetic risk factors. However, one of the risk alleles highlighted by GWAS, minor A allele with the SNP rs17319721, seems to be associated with an increased risk of GFR decline and CKD occurrence. A possible pathogenetic mechanism has been described as an increase in *SHROOM3* gene expression in the interstitial compartment mediated by a stronger association of TCF7L2/β-catenin transcriptional complex with the gene [41]. TCF7L2 factor is a downstream molecule of Wnt/β-catenin intracellular signaling pathway which regulates the expression of many genes, including *SHROOM3*. Of particular interest is the interaction of this pathway with the intracellular signaling system of TGF-β1, a key molecule in the fibrotic process. TGF-β1 increases the expression of SHROOM3 through the Wnt/β-catenin pathway, while at the same time SHROOM3 appears to facilitate intracellular signaling of TGF-β1/SMAD3 by promoting expression of genes involved in fibrosis, such as *COL1A1*. Through this interaction a positive feedback system is created that promotes intertubular fibrosis in the renal parenchyma (Fig. 3) [41].

Although A risk allele is associated with an increased risk of GFR decline and CKD, paradoxically it is also strongly correlated with lower levels of albuminuria (UACR) [42]. This discrepancy probably reflects a different effect of increased levels of SHROOM3 in the glomerular and interstitial compartments. Thus, while in the interstitial compartment A allele appears to promote renal fibrosis through increased expression of the gene, in podocytes the overexpression of *SHROOM3* appears to maintain its beneficial effect on the organization of the cytoskeleton and on the normal morphology and functionality of the podocytes and slit diaphragms, minimizing the risk of podocyte damage and the occurrence of albuminuria [39].

Alteration of the Wnt signaling pathway, a known pathway implicated in normal kidney development, is also observed with variants of the *DCDC2* gene which was prioritized recently as risk causal gene for kidney disease [22]. Recessive pathogenic variants of DCDC2 are known to cause a renal-hepatic variant of nephronophthisis-related ciliopathies that is characterized by severe early onset liver fibrosis and renal involvement with tubular defects, renal fibrosis, cyst formation and eventually CKD [43]. DCDC2 seems to regulate the canonical Wnt/β-catenin signaling pathway through a downregulation process and these loss-of-function pathogenic variants lead to an overactive Wnt signaling pathway that is known to be implicated in many kidney phenotypes such as progression of acute kidney injury to CKD, renal fibrosis in CKD, cystogenesis, podocytopathy and proteinuric CKD (Fig. 3) [44].

Finally, an intriguing category of common GWAS variants includes genes involved in known Mendelian forms of nephropathy. Some of these variants are believed to be benign confirming the fluid boundaries of the pathogenic potential of a genetic change: *ALMS1, CNNM2, CYP24A1, CACNA1S, DACH1, DCDC2, GNAS, LRP2, MUC1, RPS10, SALL1, SCARB2, SDCCAG8, SHH, SLC34A1, SLC7A9, SMAD3, UMOD, PKHD1, NPHS1, HNF1A* and *COL4A3* [19, 22, 45]. Among these genes *COL4A3* exhibits an extremely interesting genetic “behavior.” It is known that *COL4A3* rare pathogenic variants are implicated in monogenic nephropathies such as Alport syndrome and thin basement membrane disease, however a common missense genetic variant of *COL4A3* (rs55703767) has been identified recently in patients with DM type 1 displaying a protective role against the occurrence of diabetic kidney disease [44]. In contrast to the majority of GWAS variants, which act through alteration of expression level of causal genes, this variant affects the structure of the *COL4A3* gene product rather than expression levels, leading to thinner glomerular basement membrane (GBM) in individuals with DM but without causing renal damage. It seems that the thinner GBM associated with these variants (pathogenic and protective) exhibits a completely different behavior in a different context, with the thinner GBM associated with the protective variant, conferring tensile strength and flexibility to the GBM and rendering glomerulus resistant to detrimental effects of glomerular hypertension associated with diabetic kidney disease. To highlight further the complexity of the above associations, special mention can be made of rare variants, which on their own do not incur a perceptible damage that will lead to a recognizable phenotype, however when they are co-inherited on the background of another monogenic disease they exacerbate the phenotype. In the case of *COL4A* genes a representative example is the podocin variant *NPHS2*:p.Arg229Gln (rs61747728), which in homozygosity or compound heterozygosity causes an autosomal recessive form of steroid-resistant nephrotic syndrome. In heterozygosity it is associated with healthy phenotype, but when co-inherited with *COL4A3* or *COL4A4* pathogenic variants, which cause thin basement membrane nephropathy, it aggravates the phenotype resulting in development of proteinuria and progress to severe kidney function decline, and even ESRD on long follow-up. Several such variants have been identified which are viewed as genetic modifiers [46, 47].

## WHAT NEXT

GWAS have begun to expand our knowledge about the genetic basis of kidney diseases and related damage mechanisms. Beyond the obvious value of knowing the exact pathogenic pathways that are implicated in the initiation and progression of CKD, GWAS variants represent promising avenues for research in kidney diagnostics and therapeutics. The potential for the application of these genetic data both for prediction of kidney disease risk and clinical assessment of kidney outcomes has given rise to genetic risk scores that enable scientists to aggregate the individual effects of thousands of common GWAS variants in one genetic tool [48, 49]. In this way, maximization of the predictive power of GWAS results becomes feasible, permitting genetic risk stratification that can further enhance the predictive ability of traditional CKD risk models.

Furthermore, knowledge about the polygenic basis of CKD will allow a better categorization of CKD patients, and better study design and interventions in nephrology. Indeed, the heterogeneity of CKD patients has been postulated to be a major cause of the failure of many nephrology trials to demonstrate a beneficial effect [50]. For example, drugs with known beneficial effect on glomerular pathology, such as sodium-glucose cotransporter 2 inhibitors, probably show a minor effect in patients with strong genetic predisposition to CKD who harbor many common tubulo-centric risk variants, and thus they may hinder the clear benefit for those patients whose main pathophysiological disturbance is glomerular hyperfiltration. Logically, the integration of more information about CKD patients’ status (etiology, genetic background, underlying pathophysiological mechanisms, comorbidities, etc.) during the allocation process will overcome these barriers, leading to better trial design and improved patient outcomes.

Last but not least, we know with great certainty that CKD is a complex disease entity with a high diversity of molecular disease drivers and up to 100 biological pathways involved in the pathophysiology [51]. The lack of understanding of kidney biology underpinning disease is a major cause of the scarcity of specific therapeutic interventions for CKD, rendering future drug discovery particularly challenging. GWAS genetic information attempts to bridge this gap by revealing new and valid drug targets in order to optimize drug therapy and make some progress in halting or reversing kidney disease progression. The future of nephrology is a new patient-centric approach, and GWAS and related methodologies are very powerful tools by which to achieve it.

## Data Availability

No new data were generated or analyzed in support of this research.
